# Neurosyphilis Reemergence in the Modern Day: A Case Report

**DOI:** 10.7759/cureus.110409

**Published:** 2026-06-07

**Authors:** Christopher G Rohner, Andrew Trom

**Affiliations:** 1 Department of Emergency Medicine, Inspira Health, Philadelphia, USA; 2 Department of Emergency Medicine, Inspira Medical Center Mullica Hill, Mullica Hill, USA

**Keywords:** atypical syphilis, lumbar puncture, neurosyphilis, rpr, syphilis

## Abstract

Syphilis, once considered a disease in decline, has re-emerged as a significant public health concern in the United States. This case report presents the clinical course of a 42-year-old male with a rare presentation of neurosyphilis. The patient presented twice to the ER within 60 days with vague intermittent symptoms, including numbness/tingling of his hands and feet, which the patient initially ignored. This progressed into balance issues, tinnitus, difficulty focusing, inattentiveness, and behavior changes. Family states he had started behaving very oddly, had wax-and-waning confusion, and also had a moment where he was hallucinating. The patient's first evaluation in the ER was roughly three weeks prior to the second visit. The workup included a CT of the head, which was negative, among other tests for toxic/metabolic causes. After a grossly unremarkable workup at the initial visit, the patient was further discharged at that time. At home, the patient stated his symptoms continued, he followed up with his PCP as he was directed, and the PCP ordered a rapid plasma reagin (RPR) test for continued symptoms and found the patient to be RPR (+). The patient then presented to the ED for a second evaluation sent by PCP with a RPR (+), continued symptoms, and requested further evaluation. On a more comprehensive social history investigation, the patient reported a painless penile lesion roughly three years ago after a sexual encounter. The lesion went away on its own, and the patient never sought treatment for the lesion. On the second ER presentation, the patient then had a more in-depth workup, including lumbar puncture results, which showed suspicion of neurosyphilis. The patient was then admitted with disposition for treatment of neurosyphilis. While admitted to the hospital, the patient was reported as remaining stable, clinically improved, and was appropriately treated for neurosyphilis with 14 days of IV antibiotics. The patient was in the hospital for a total of a week and then discharged home with IV antibiotics set up for home completion of the 14-day course for the treatment of neurosyphilis.

## Introduction

Syphilis is a sexually transmitted condition caused by the spirochete Treponema pallidum [1]. The pathogenesis is characterized by multiple stages that present in several temporal stages. Declining incidences in the modern day have also caused testing to decline in practice, but due to recent social trends, syphilis has seen a resurgence in parts of the Americas [2]. The initial phase of primary syphilis is characterized by a primary lesion (chancre), which appears after 2-6 weeks of incubation. This is typically described as a single, painless ulcer-like lesion. Some present with multiple lesions, although atypical with regional painless adenopathy. This typically appears at the point of contact, including the penis, rectum, mouth, external genitalia, cervix, or labia, and heals in 4-6 weeks. Secondary syphilis, which appears 6-8 weeks after the healed chancre lesion, is characterized by mucocutaneous lesions and lymphadenopathy. Skin lesions are typically maculopapular, reddish to pink, non-pruritic, and spread about the extremities, later involving the palms and soles. Atypical presentations include healing chancre that may still be present in a minority of patients, and stages that overlap more frequently in HIV patients. Generalized symptoms have also been presented with fever, malaise, headache, and weight loss. Also noted that in this stage, CSF abnormalities can begin to be detected; however, CNS involvement can be symptomatic or asymptomatic. Secondary syphilis transitioning into latent syphilis can present with mucocutaneous lesions or be asymptomatic, which is a challenge. At this time, serologic testing, including fluorescent treponemal antibody-absorption and Treponema pallidum particle agglutination test, can be used to detect, until further symptoms are present, but screening may or may not occur. A hallmark that characterizes tertiary syphilis is the presence of more overt signs, such as CNS manifestations, and hence the description of neurosyphilis. More commonly seen in HIV patients, neurosyphilis can present months to years later [3-5]. Meningeal signs include headache, vomiting, neck stiffness, vertigo, cranial nerve involvement, seizures, altered mental status, psychological abnormalities, uveitis, iritis, and hearing loss. Meningovascular signs include meningitis with inflammatory vasculitis leading to stroke syndrome. Parenchymal signs include paresis, hyperactive reflexes, Argyll Robertson pupils, hallucinations, memory defects, speech changes, tabes dorsalis, gait ataxia, foot drop, paresthesias, bladder dysfunction, impotence, and areflexia. The preferred mode of testing at this stage currently is CSF-VDRL [1]. It is noted that rapid plasma reagin (RPR) titers of >1:32 are at higher risk of having neurosyphilis, with a lower threshold for those with HIV. Due to the extensive modes of presentation and symptoms, the differential can be wide, and clinical diagnoses have been considered when clinically appropriate. As this presentation can be challenging to diagnose in the ER setting, coupled with the rise in social trends, screening for syphilis may become a more common practice in the ER moving forward.

## Case presentation

**Figure 1:**
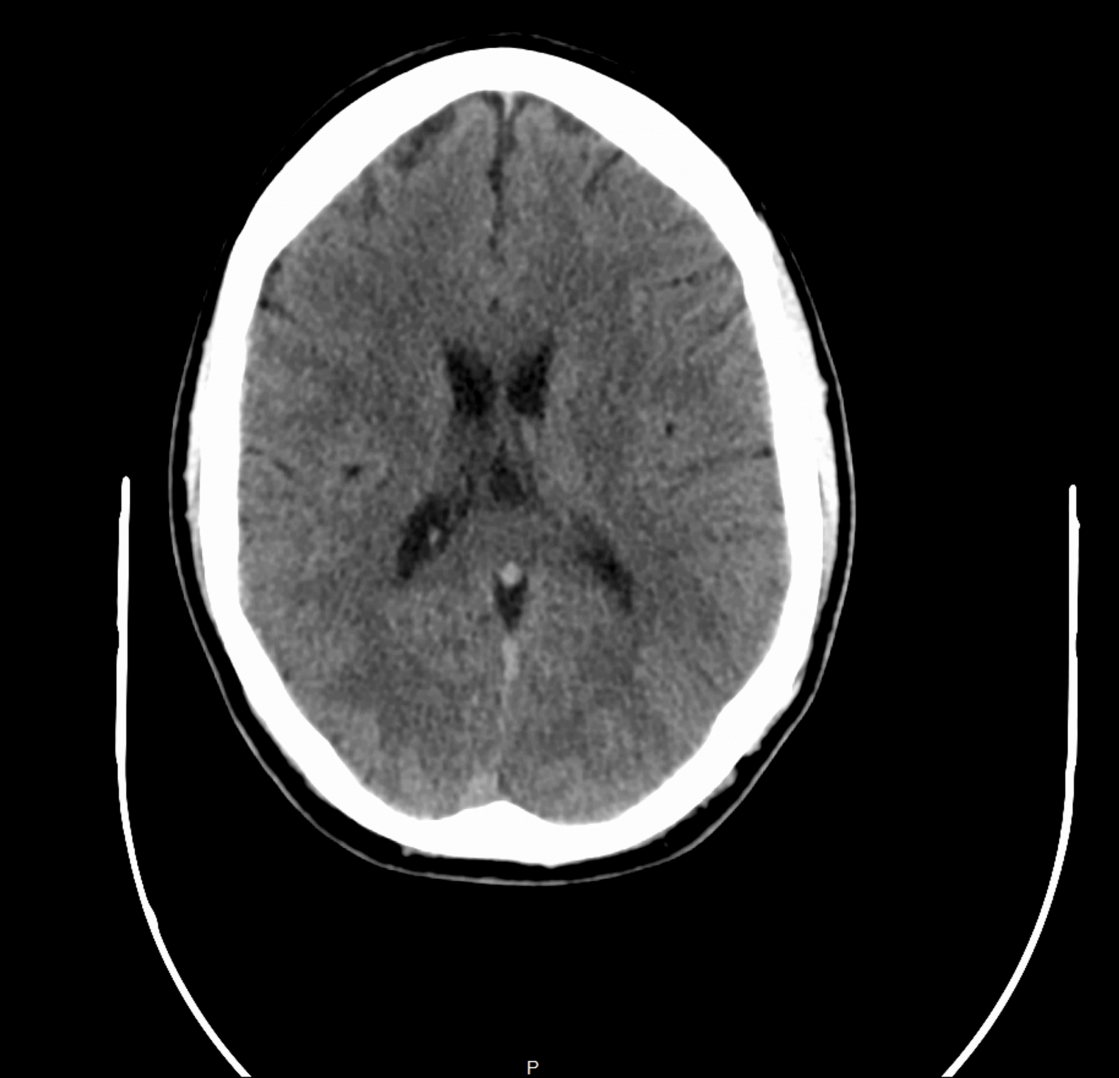
CT head without contrast (axial view)

**Figure 2:**
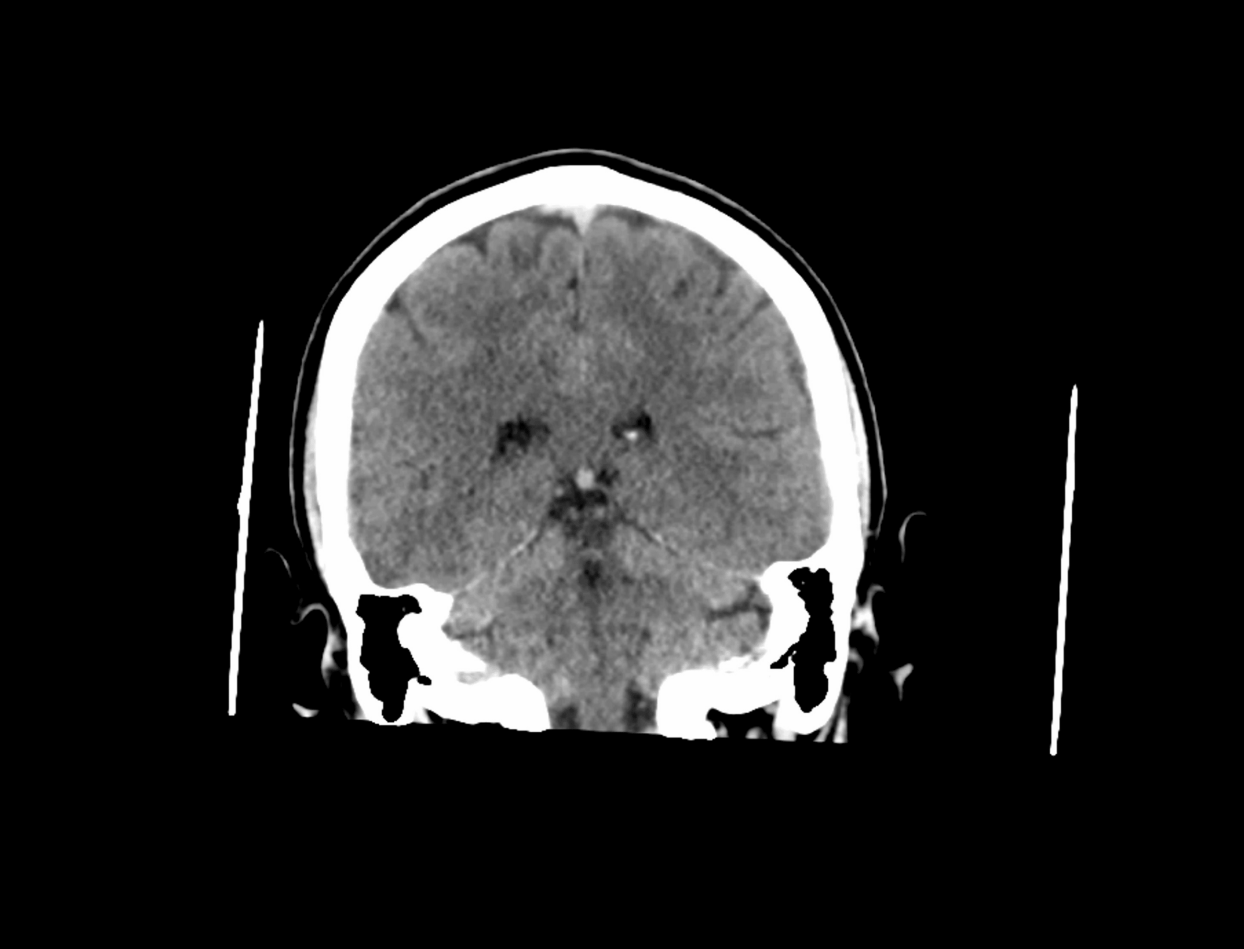
CT head without contrast (coronal view)

A 42-year-old male presented to the ER with suspicion of a rare presentation of neurosyphilis. The patient presented for his second ER visit with two months of vague on-off symptoms, including neuro complaints such as paresthesias, tinnitus, confusion, and behavioral complaints, as well as psychiatric complaints such as hallucinations. He denied any past medical or psychiatric history initially but did report a painless chancre on his genitals three years ago after a sexual experience. The patient did not seek treatment for the lesion, and it resolved on its own. His physical exam at that time showed normal vitals and a male in no acute distress. He was slow to speak with a blunted affect, otherwise with a non-focal neuro exam at the time of ED evaluation. Diagnostic workup included CBC, BMP, LFT, Hepatitis screen, TSH, Drug screen, folate, vitamin B12, urinalysis, HIV, HSV, RPR, and viral respiratory swab. Lumbar puncture was performed, and CSF studies including VDRL were performed. Grossly unremarkable images of the CT head without contrast are shown in Figures 1, 2. CT head without contrast of the head showed no pertinent positives. Pertinent positive results are listed in Table 1.

**Table 1:** Immunology/hepatitis/viral results

Laboratory Test	Result	Reference Range/Interpretation
Hepatitis B Core Antibody Total (Hep B C Ab Tot)	Negative	Negative
Hepatitis B Surface Antibody Quantitative (Hep B S Ab IM Qn)	4.1 (L)	<10 mIU/mL: Non-immune / low immunity
Hepatitis B Surface Antigen (Hep BS Ag)	Negative	Negative
Hepatitis C Antibody (Hep C Ab)	Non-Reactive	Non-reactive
HCV Interpretation	Non-Reactive	Non-reactive
Rapid Plasma Reagin (RPR)	Reactive (+)	Non-reactive
RPR Quantitative	1:16 (H)	Non-reactive; elevated titer consistent with active infection
RPR + Treponemal Antibody Interpretation	Reactive (+)	Non-reactive
SARS-CoV-2	Negative	Negative
Influenza A	Negative	Negative
Influenza B	Negative	Negative
Respiratory Syncytial Virus (RSV)	Negative	Negative
HSV-1 IgG	Non-Reactive	Non-reactive
HSV-2 IgG	Reactive (+)	Non-reactive
HIV	Not Reactive	Non-reactive

## Discussion

As suggested by recent trends, syphilis has re-emerged as a significant public health concern in the United States. According to the Centers for Disease Control and Prevention (CDC), rates of primary and secondary syphilis have increased significantly over the past decade. Reported cases of primary and secondary syphilis in the United States increased by more than 70% between 2017 and 2023, highlighting the growing public health burden of this infection [2].

This resurgence makes recognition of atypical and delayed presentations particularly important for emergency physicians. Neurosyphilis has historically been associated with untreated late-stage disease and immunocompromised states, particularly HIV co-infection. However, emerging literature demonstrates that central nervous system involvement may occur during any stage of infection and in immunocompetent individuals [3,4]. Our patient represents an increasingly relevant but atypical presentation: an immunocompetent individual with no known prior diagnosis of syphilis presenting with subacute neuropsychiatric changes. In this case, the predominant features were waxing and waning confusion, personality change, hallucinations, inattentiveness, and functional decline. These symptoms initially triggered a psychiatric concern rather than an infectious evaluation. Notably, the neurologic exam was largely non-focal, and the initial ED workup including CT imaging was unrevealing. Increasing numbers of case reports and retrospective studies describe patients presenting primarily with psychiatric or cognitive symptoms rather than focal neurologic deficits, often resulting in delayed diagnosis and treatment [5]. One of the most clinically relevant aspects of this case is the negative CSF-VDRL in the presence of elevated CSF protein and positive serum RPR (1:16). The CDC notes that CSF-VDRL remains highly specific but relatively insensitive for neurosyphilis, and therefore, a negative CSF-VDRL does not exclude the diagnosis in patients with supportive clinical findings and CSF abnormalities [6]. A negative result does not exclude neurosyphilis in the appropriate clinical context. 

Given these findings, the decision to treat neurosyphilis despite a negative CSF-VDRL was considered appropriate treatment as the multi-disciplinary decision ultimately continued IV Penicillin G daily for neurosyphilis, and the patient continued treatment on discharge for 14 days and was set up with home treatments with IV Penicillin G. For EM providers, this reinforces that neurosyphilis remains in part a clinical diagnosis supported by serology and CSF abnormalities despite a negative CSF-VDRL. Some challenges and limitations, however, include a lack of long-term follow-up to assess cognitive recovery after treatment.

## Conclusions

This case highlights the rising trends of neurosyphilis as a clinically relevant diagnosis in modern emergency medicine. In an era of increasing syphilis incidence, emergency physicians must consider atypical presentations. Such atypical presentations can be neurosyphilis that occur in immunocompetent individuals, who may present without focal neurologic deficits, and cannot be excluded solely by a negative CSF-VDRL. Subacute cognitive and behavioral changes warrant inclusion of syphilis in the diagnostic workup, particularly when social history or sexual risk factors are present. This case reinforces the importance of broad differentials and thorough social history and avoidance of premature diagnostic closure in patients with unexplained neuropsychiatric decline. As syphilis continues to occur, maintaining a high index of suspicion may prevent missed opportunities for intervention in this population and potentially reversible disease for patients and ultimately improve outcomes.
